# Behavioral outcome prediction among children using machine learning

**DOI:** 10.6026/973206300211555

**Published:** 2025-06-30

**Authors:** Samir P.V, Aruna Kumari G, Nandini Biradar, Kodali Srija, Debasmita Das, Sukabhogi Anusha

**Affiliations:** 1 Department of Pedodontics & Preventive Dentistry, Kalinga Institute of Dental Sciences, KIIT Deemed to be University, Bhubaneswar-751006, India; 2 Department of Pedodontics & Preventive Dentistry, Malla Reddy Dental College for Women- Mallareddy Viswavidyapeeth, Hyderabad, India; 3 Department of Conservative dentistry and Endodontics, Private Practitioner, Biradar Dental care, Bidar, Karnataka, India; 4 Department of Pedodontics and Preventive Dentistry, Hi-Tech Dental College & Hospital, Bhubaneswar, India; 5 Department of Pedodontics and Preventive Dentistry, Meghana Institute of Dental Sciences, Nizamabad- 503001, Telangana, India

**Keywords:** Pediatric dentistry, machine learning, behavioral prediction, dental anxiety, random forest, frankl scale

## Abstract

Behavioural management in paediatric dentistry is essential for treatment success, yet predicting a child's behavior remains a challenge. This study used machine learning models on data from 120 children aged 4-10 years, incorporating clinical and historical variables such as age, dental history and parental anxiety. Among the models tested, Random Forest achieved the highest accuracy (87.5%) in predicting behavior based on the Frankl scale. Key predictors of negative behavior included younger age, high parental anxiety and prior negative dental experiences. These findings highlight the potential of machine learning to support behavior guidance planning and improve clinical outcomes.

## Background:

The accurate prediction of paediatric cognitive and behavioural outcomes remains a significant challenge in clinical and developmental neuroscience. Traditional diagnostic pathways often fail to identify high-risk children early enough for effective intervention. Recent advancements in machine learning (ML) and big data analytics have shown promising potential in addressing this gap through predictive modelling across a range of paediatric conditions [1, 2]. Several studies have demonstrated the utility of ML in predicting behavioural and neurodevelopmental outcomes using diverse data inputs including clinical, behavioural, physiological and socio-demographic variables. For instance, ML algorithms using electronic health records have effectively predicted clinical outcomes during emergency triage in children, outperforming conventional tools in risk stratification and care planning [2]. Similarly, ML approaches have successfully identified behavioural indicators for ADHD [3], disruptive behaviour via physiological smart-watch data [4] and future onset of bipolar disorder based on childhood behavioural features [5, 13]. In pediatric traumatic brain injury, ML models have exceeded the accuracy of CT-based systems in predicting long-term neurological outcomes [6, 12]. Autism spectrum disorder (ASD) has also been a focal point, with studies showing that ML can reliably forecast treatment responses and behavioural challenges based on sensory processing and executive function profiles [7, 8-9, 15]. In psychiatric contexts, ML has predicted suicide risk [10], post-traumatic stress [14] and outcomes from internet-based CBT in obsessive-compulsive disorder [11].

Building on these applications, researchers have explored the use of ML to predict cognitive development trajectories by incorporating neurocognitive assessments, genetic data and environmental exposures. Behera *et al.* [1] utilized an interdisciplinary dataset comprising socioeconomic factors, parental history and cognitive testing outcomes, demonstrating that predictive models could identify children at risk for poor cognitive performance well before clinical symptoms manifest. The integration of such heterogeneous data types improves the robustness of predictive models, ensuring higher sensitivity and specificity in developmental risk profiling. Moreover, ML models have also shown superiority over traditional statistical approaches in intervention outcome prediction. In a quasi-experimental setting, Sun *et al.* [8] demonstrated that ML techniques, particularly support vector machines, predicted which children with autism would benefit most from structured motor skill interventions such as mini-basketball training. These findings suggest that ML can not only support diagnostic predictions but also enhance personalized treatment planning by identifying likely responders to specific therapies. As data availability and computational power continue to increase, ML holds transformative potential to shift pediatric care from reactive to proactive by offering early, data-driven insights into neurodevelopmental and behavioural disorders.

## Materials and Methods:

## Study population:

The study included 120 children aged between 4 and 10 years who reported for routine dental treatment. Children with special healthcare needs or those on medications affecting behavior or cognition were excluded. Sampling was done using a non-probability convenience sampling method.

## Data collection:

Data were collected through a structured form consisting of the following variables:

[1] Demographic details: age, gender

[2] Dental history: number of previous dental visits, experience during past dental treatment (positive/negative)

[3] Parental factors: anxiety level measured using a Modified Corah's Dental Anxiety Scale

[4] Clinical indicators: caries index (DMFT/deft), oral hygiene status (Simplified Oral Hygiene Index) and subjective pain perception using a Faces Pain Scale

## Behavioral assessment:

Child behavior during the dental procedure was assessed using the Frankl Behaviour Rating Scale, which classifies behavior into four categories: definitely negative, negative, positive and definitely positive. This assessment was conducted by a calibrated examiner who was blinded to the machine learning process.

## Machine learning model development:

Data were pre-processed by handling missing values and converting categorical variables into numerical formats using one-hot encoding. The dataset was split into a training set (80%) and a testing set (20%). Three supervised machine learning algorithms were implemented:

[1] Decision tree

[2] Random forest

[3] Support vector machine (SVM)

These models were developed using Python and the Scikit-learn library. The models were trained using the training dataset and evaluated on the testing set. The outcome variable was the child's behavioral response (binary classification: positive vs. negative behavior).

## Model evaluation:

Performance of the models was evaluated using standard classification metrics:

[1] Accuracy = (True Positives + True Negatives) / Total Predictions

[2] Precision = True Positives / (True Positives + False Positives)

[3] Recall (Sensitivity) = True Positives / (True Positives + False Negatives)

[4] F1-score = 2 x (Precision x Recall) / (Precision + Recall)

The model with the highest overall performance across all metrics was identified as the most effective for predicting behavioral outcomes.

## Results:

## Baseline characteristics:

A total of 120 children were included in the study, comprising 68 males (56.7%) and 52 females (43.3%). The mean age was 6.7 ± 1.9 years. A majority of participants (62.5%) had at least one prior dental visit and 41.6% reported a negative past dental experience. High parental anxiety scores were observed in 39.2% of cases. The distribution of clinical variables and behavioral outcomes is presented in Table 1 (see PDF). As shown in Table 1 (see PDF), 39.2% of children exhibited negative behavior during treatment. A higher proportion of negative behavior was observed among younger children, those with high parental anxiety and those reporting previous negative dental experiences.

## Machine learning model performance:

Among the three models evaluated, the Random Forest classifier demonstrated the highest predictive accuracy. Performance metrics for all models are detailed in Table 2 (see PDF). As seen in Table 2 (see PDF), the Random Forest model achieved the highest accuracy (87.5%) and F1-score (85.1%), followed by the SVM model with an accuracy of 81.3%. The Decision Tree model showed moderate performance across all metrics. Key variables contributing to accurate predictions in the Random Forest model included age, parental anxiety and prior dental experience.

## Discussion:

The results of this study reinforce the growing evidence that machine learning (ML) tools, particularly ensemble methods like Random Forests, can significantly enhance the prediction of behavioural outcomes in paediatric dental settings. The observed superiority of the Random Forest model across accuracy, precision, recall and F1-score echoes prior findings from broader pediatric research domains, where similar methods have consistently outperformed simpler algorithms such as decision trees or logistic regression [1, 2 and 13]. In our study, key variables influencing behavioural prediction-age, parental dental anxiety and prior dental experience-align closely with earlier investigations into behavioural and cognitive predictors across various pediatric conditions. For instance, younger age has been linked to behavioural deregulation and reduced procedural cooperation, a trend echoed in ADHD prediction using behavioural activity data by Maniruzzaman *et al.* [3] and in autism-related disruptive behavior prediction using physiological data by Romanowicz *et al.* [4]. The strong predictive power of parental anxiety is also well-established, with studies confirming its direct impact on child distress levels in clinical settings [6]. Importantly, our findings resonate with those of Behera *et al.* [1], who emphasized that incorporating multidimensional inputs such as familial, behavioural and clinical features leads to highly accurate cognitive outcome predictions in children. Although their study focused more broadly on cognitive development, the integration of socio-environmental and clinical variables mirrors the structured feature set used in the present research. Similarly, the wearable-derived biomarkers used by Romanowicz *et al.* [4] in predicting disruptive behavior indicate a shift toward real-time and multimodal behavioural prediction models, suggesting future directions for expanding behavioural data collection in dental clinics. This study's focus on using ML within a dental context distinguishes it from many prior works that emphasized neurological or psychiatric outcomes [5, 6, 10 and 11]. Nevertheless, the observed pattern-that ML models can handle complex variable interactions more effectively than conventional tools-is consistent with findings in pediatric traumatic brain injury prognosis [6, 12] and suicide risk stratification [10]. Our Random Forest model's robustness further reflects the value of using ensemble learning in healthcare, particularly where outcomes are influenced by interacting clinical and psychosocial variables. Unlike many previous works that used outcomes from long-term follow-up or neuroimaging, this study employed the Frankl Behaviour Rating Scale-a validated yet practical tool for chairside behaviour classification [14]. While the binary classification (positive vs. negative) simplifies modelling, it may not capture the full behavioural nuance observed during dental procedures. Studies like that by Lenhard *et al.* [11] on OCD treatment outcomes demonstrate the utility of continuous outcome modelling, which may benefit future pediatric dental ML applications as well. Furthermore, predictive modelling in ASD therapy outcomes, such as Sun *et al.*'s work with motor skill interventions [8], reveals the promise of ML in intervention stratification. Our findings similarly support the use of behavior prediction for individualized treatment planning in dental care-where interventions like tell-show-do, parental counselling, or pharmacologic behavior control can be proactively applied based on predicted cooperation levels. The relatively high performance of all three tested ML models also emphasizes the importance of using cross-validated, well-tuned models even in modestly sized datasets. However, like previous studies limited by sample diversity or follow-up length [9, 12 and 15], our study's single-centre design and limited inclusion of psychosocial and environmental features (*e.g.*, parenting style, education level, or temperament) highlight areas for expansion. Longitudinal data and real-time digital behavior tracking, as explored in Saxe *et al.*'s PTSD prediction work [14], may provide more robust and dynamic predictive frameworks for future dental settings.

## Conclusion:

Machine learning models, particularly Random Forests, show strong potential in predicting pediatric dental behavior using routine clinical and historical data. Early identification of children at risk for negative behavior can enhance treatment planning and improve patient cooperation, making AI-driven tools valuable assets in pediatric dental practice.

## References

[1] Behera N.R (2021). In 4th International Conference on Innovative Practices in Technology and Management (ICIPTM)..

[2] Goto T (2019). JAMA Netw Open..

[3] Maniruzzaman M (2022). Appl Sci..

[4] Romanowicz M (2023). J Child Adolesc Psychopharmacol..

[5] Uchida M (2022). J Psychiatr Res..

[6] Hale A.T (2018). Neurosurg Focus..

[7] Alateyat H (2022). Front Mol Neurosci..

[8] Sun Z (2022). Int J Ment Health Promot..

[9] Usta M.B (2019). Psychiatry Clin Psychopharmacol..

[10] Su C (2020). Transl Psychiatry..

[11] Lenhard F (2018). Int J Methods Psychiatr Res..

[12] Tunthanathip T (2021). Chin J Traumatol..

[13] Uchida M (2022). J Psychiatr Res..

[14] Saxe G.N (2017). BMC Psychiatry..

[15] Linstead E (2015). IEEE 14th International Conference on Machine Learning and Applications (ICMLA)..

